# Unbiased Characterization of Peptide-HLA Class II Interactions Based on Large-Scale Peptide Microarrays; Assessment of the Impact on HLA Class II Ligand and Epitope Prediction

**DOI:** 10.3389/fimmu.2020.01705

**Published:** 2020-08-05

**Authors:** Mareike Wendorff, Heli M. Garcia Alvarez, Thomas Østerbye, Hesham ElAbd, Elisa Rosati, Frauke Degenhardt, Søren Buus, Andre Franke, Morten Nielsen

**Affiliations:** ^1^Genetics & Bioinformatics, Institute of Clinical Molecular Biology, Christian-Albrechts-University of Kiel, Kiel, Germany; ^2^IIBIO, UNSAM-CONICET, Buenos Aires, Argentina; ^3^Department of Immunology and Microbiology, University of Copenhagen, Copenhagen, Denmark; ^4^Department of Health Technology, Technical University of Denmark, Lyngby, Denmark

**Keywords:** ultra-high density peptide microarray, MHC class II, HLA, antigen presentation, prediction, peptide binding, high-throughput, machine learning

## Abstract

Human Leukocyte Antigen class II (HLA-II) molecules present peptides to T lymphocytes and play an important role in adaptive immune responses. Characterizing the binding specificity of single HLA-II molecules has profound impacts for understanding cellular immunity, identifying the cause of autoimmune diseases, for immunotherapeutics, and vaccine development. Here, novel high-density peptide microarray technology combined with machine learning techniques were used to address this task at an unprecedented level of high-throughput. Microarrays with over 200,000 defined peptides were assayed with four exemplary HLA-II molecules. Machine learning was applied to mine the signals. The comparison of identified binding motifs, and power for predicting eluted ligands and CD4+ epitope datasets to that obtained using NetMHCIIpan-3.2, confirmed a high quality of the chip readout. These results suggest that the proposed microarray technology offers a novel and unique platform for large-scale unbiased interrogation of peptide binding preferences of HLA-II molecules.

## Introduction

The highly diverse major histocompatibility complex (MHC) proteins play a major role in the adaptive immune system. MHC class II proteins present peptides of variable lengths mainly derived from extracellular antigens (1). In humans, MHC is called human leukocyte antigen (HLA). The HLA locus is highly polymorphic, resulting in different HLA molecules having a specific peptide binding preference and specific peptidomes. The HLA is an important susceptibility locus in genetic studies of many immune-related diseases, often with multiple HLA alleles playing a role (2–4). However, beyond the suggested association, these studies do not inform about the causes of a disease, i.e., the antigen/epitope that binds to associated HLA proteins and potentially drive the disease onset. To make this link between HLA and antigen, further studies to characterize the peptidome bound by specific HLAs are necessary (5, 6). To this end, efficient and reliable high-throughput technologies for measuring peptide-HLA interaction are needed. Different assay types may be used to record the interaction between HLA and peptides (7). Classical *in-vitro* assays measure one single interaction of a synthetic peptide and an HLA-molecule in one experiment. Mass spectrometry of HLA eluted peptides considers the whole process of antigen synthesis up to presentation might fail the identification of low abundant peptides or modified peptides. To avoid costs and time delays *in-silico* prediction tools for HLA binding and antigen presentation have been trained on measured assay data (8–17).

Here, we set out to overcome the experimental limitations outlined above by employing our high-density peptide microarray data, a new high-throughput *in-vitro* technology (18, 19), combined with synthetic *in-vitro* generated HLA-II molecules (20) to perform large-scale unbiased characterization of HLA-II allele-specific binding. Earlier work has used peptide microarray for measuring peptide-HLA interaction, but this was limited to thousands of peptides per array (21). Here, the high-density peptide microarray enables the *in-situ* synthesis of over 2 million peptides per array on about 2 cm^2^ (18). To this end, we synthesized about 70,000 random peptides in triplicates on one array, allowing us to generate vastly more data points than the combined number of all HLA-DR epitopes registered in the immune epitope database IEDB (www.iedb.org) (7). This technology enables the analysis of whole proteomes of interest in one single experiment and the systematical analysis of post-translational modifications. Our presented technology offers a unique solution to produce large datasets to characterize binding properties of HLA-II molecules and improve the *in-silico* prediction of peptide-HLA interaction while being suitable for hypothesis driven tests.

To prove the quality of the high-density peptide microarray for characterizing peptide-HLA-II interactions, we selected four HLA-DRB1 proteins that are known to be strongly associated with ulcerative colitis, a complex chronic inflammatory bowel disease (2). For DRB1^*^01:03, DRB1^*^03:01, DRB1^*^15:01, and DRB1^*^15:02, a set of 69,815 random peptides were analyzed. To mine the extracted datasets and to learn predicting peptide-HLA binding, we applied NNAlign (22), as well as a deep learning approach, referred to as PIA (Peptide Immune Annotation). Using the obtained models, we assessed the quality of the chip readout in terms of identified binding motifs, and power to predict publicly available MS data from elution experiments as well as CD4+ epitope datasets in comparison to NetMHCIIpan-3.2 (8).

## State of Research

### Peptide-HLA Assays

The IEDB collects all types of immune epitopes. The oldest record is from 1952 (7). From the 90s to 2010, *in-vitro* assays measuring binding of synthetic peptides to HLA molecules (20, 23) were the most common MHC binding assays (www.iedb.org). In the last 5 years, mass-spectrometry (MS) sequencing of HLA eluted peptides (24, 25) became more popular (first records already in 1991).

Both methods have their strengths and weaknesses. *In-vitro* binding studies can measure interaction of individual peptide-HLA combinations. However, this approach is highly cost-intensive (one assay per peptide) and the assay fails to address some events leading up to effective HLA antigen presentation such as antigen processing, the effects of chaperones like HLA-DM, editing of the repertoire of HLA bound peptides (12, 14), and HLA-peptide complex stability. In contrast, recent advances in MS technology have expanded the detectable peptide repertoire presented by HLA molecules (immunopeptidome) by use of liquid chromatography MS. Immunopeptidome data include comprehensive information on the complex HLA ligand presentation (26), and analysis results of such data are a rich source of information for learning about the underlying rules of HLA antigen presentation. However, MS HLA peptide elution data mainly covers self-peptides and is assumed to miss low abundant peptides (26), further post-translational modifications might be identified but misinterpreted (27). Another problem arising with natural cell lines is that they most often present different HLA proteins. To solve this problem, either homozygous cell lines, tagging of a specific HLA allele (14, 28) or algorithms for deconvolution of the HLA proteomes can be employed (16, 17, 29). However, deconvolution has been shown to be of limited success in cases of lowly expressed HLA proteins or cells expressing HLA proteins with overlapping proteome specificity (14).

### Peptide-HLA Binding Prediction

Beyond the different experimental approaches developed to specify peptide-HLA interaction, large efforts have been made to develop prediction models capable of accurately predicting peptide-HLA binding. Historically, most *in-silico* methods have been developed based on *in-vitro* binding data and an exemplary state-of-the-art computational method is NetMHCIIpan (8, 9, 30). Recently, prediction methods have been developed from HLA-II elution data (10–15). The results suggest that the inclusion of elution data has a positive impact on the predictive power of *in-silico* methods in particular for the prediction of HLA antigen presentation (10–15). Algorithms can be trained on either *in-vitro* or *in-vivo* data (8, 11, 14, 16), but a benefit from training on the two data types combined has been reported (10, 12, 13, 15, 17). However, currently even the best methods for prediction of HLA-II binding and antigen presentation suffer from an excessive number of false positive predictions.

## Materials and Methods

### Microarray

Peptide microarrays were produced by Schafer-N (Copenhagen, Denmark). Briefly, a Nexterion E microscope slide (Schott, Jena, Germany) were amino functionalized with a 1% w/v linear copolymer (1T0C) of N,N-dimethylacrylamide (Sigma-Aldrich) and aminoethyl methacrylate (Sigma-Aldrich) and used as substrate for solid-phase peptide synthesis. The peptide synthesis was initiated with the coupling of one unit of epsilon-amino-capronic acid (EACA) followed by the peptide sequences. The 1T0C and EACA unit served as a spacer between the array surface and the peptides allowing the HLA class II molecules to interact and peptides to protrude out of the HLA in both ends. For each experiment the same array-design was chosen. The peptide chips were subdivided into 12 sectors with a marker peptide “PVSKMRMATPLLMQA” of the HLA-II antigen gamma chain (CD74; UniProt: P04233-1: 103-117) placed multiple times in all the sectors corners. 69,815 different random natural 13-mer peptides were placed on the chip in triplicates. The chip does not contain peptides containing more than four poly residues (e.g., RRRR) as poly residues are difficult to synthesize and have a tendency toward unspecific binding.

Peptide microarrays were incubated with different HLA-DR molecules as previously described (31). Briefly, HLA-DR molecules were diluted from a stock (8 M Urea, 25 mM Tris, pH8) to achieve a final concentration of 500 nM HLA-DR in PBS, 0.05% Lutrol F68, 20% Glycerol pH 7.4 and added (overlaid) to the peptide array surface and allowed to fold for 24 h at 18°C before washing and staining with monoclonal mouse anti-HLA-DR (L243) and goat anti-mouse-Cy3. The peptide arrays were scanned with a laser-scanner (InnoScan, Innopsys, France) at a resolution of 1 μm and the amounts of bound HLA-DR were quantified to intensities between 0 and 254 by a proprietary software (Peparray, Schafer-N, Denmark). Larger spots with high values were excluded as noise.

The data was normalized by taking the median intensity of each repeated measurement $\tilde{x_{i}}$ for each peptide and transformed to fall in the range 0–1 by $\frac{log\left(\tilde{x_{i}}+1\right)}{log\left(max\left(\tilde{x}\right)+1\right)}$. The data was split into one test dataset comprising 10% of the data and a 10-fold cross-validation dataset of the remaining data. To ensure limited data redundancy between subsets, therefore similar peptides [e.g., a 9-mer overlap (underlined amino acids in the following are the same), for example AALITRGLTEMGR and ARTALITRGLTEM, or at least 11 of the 13 amino acids in the same order, for example ADLGSGAGAAGLA and ALGSGAAGAAFGL] were placed into the same subset. For the performance evaluation, the data was back-transformed to the intensity scale.

### Consistency Metrics

We evaluated the consistency of the triplicates using the coefficient of variation (CoV) and the Pearson Correlation Coefficient (PCC) between three replicates. The CoV for each repeated peptide measurement was calculated as the standard deviation of intensity divided by mean intensity +1 and the mean CoV over the 69,815 peptides for all four alleles was given.

The PCC between the three replicates was calculated combining the pairwise PCCs *R*_12_, *R*_13_, and *R*_23_ as *R*_123_=$\sqrt{{R_{12}}^{2}+{R_{13}}^{2}+{R_{23}}^{2}-2^{*}\left(R_{12}^{*}R_{13}^{*}R_{23}\right)}$ (32).

### Epitope and Eluted Ligand Test Datasets

T cell epitopes and HLA ligands obtained from mass spectrometry were downloaded from IEDB and used as independent test data (www.iedb.org, June 18th 2019) (7). Only positive linear peptides with a length between 13 and 19 amino acids were used. Data with an overlapping sequence of at least 9 amino acids with the peptide microarray data or an unknown amino acid were excluded. This resulted in 502 epitopes and 719 ligands for the four alleles (Supplementary Table 1).

Negative data (peptides thought not to bind the respective alleles) were added by downloading the sequence of the epitope/ligand source protein as linked by IEDB from NCBI (www.ncbi.nlm.nih.gov), and *in-silico* digesting by a sliding window of the length of the ligand/epitope into overlapping peptides. Peptides with an overlap of 9 amino acids with the peptides used in training or the positive peptides were excluded.

For predicting the binding affinity for a peptide, prediction on all 13-mer subsequences was made and the highest prediction value reported.

Finally, the performance for each epitope/ligand was reported as the Frank value. The Frank value of a binding peptide is the ratio of the number of peptides with a higher predicted binding score in the source protein divided by the overall number of peptides within the protein (8).

### NNAlign

NNAlign-2.1 was used on the peptide microarray data (22). NNAlign generates artificial neural network models of receptor-ligand interactions. The program takes as input a set of ligand sequences with target values; it returns a sequence alignment, a binding motif of the interaction, and a model that can be used to scan for the motif in other sequences. Further details of the used parameters can be found in the Supplementary Methods. The motifs generation by Seq2Logo (33) is automatically performed by NNAlign.

### Deep Learning Model PIA

PIA is a gated recurrent neural network (GRU) based model (34) implemented using Keras (https://keras.io) deep learning framework with TensorFlow (www.tensorflow.org). Further details on the model architecture can be found in the Supplementary Methods.

For generating the logos, 500,000 13-mers were randomly selected from the human reference proteome and screened using PIA. The top 1% of peptides were submitted to GibbsCluster-2.0 (29) for motif identification.

## Results

High-density peptide microarrays were used to identify large, unbiased peptidome datasets for four HLA-DR molecules. We describe the raw peptide chip readout to quantify data consistency and make comparisons to earlier *in-vitro* binding experimental results. Further, we describe the results of applying two machine-learning frameworks to mine and extract the rules for peptide-HLA binding from the chip data, and we assess the quality of the chip data by comparing the power of the constructed models to that of NetMHCIIpan-3.2 for prediction of HLA ligands and epitopes.

### Microarray Experiments

The peptide microarray contained 69,815 random 13-mer peptides. An example of the raw readout of the array is shown in Supplementary Figure 1, confirming overall clear signals corresponding to discrete peptides. To assess the accuracy and consistency of the array readout, two metrics were used: the CoV and correlation coefficient between the three repeated peptide measurements (for details see **Materials and Methods**). Overall, this analysis demonstrated highly consistent values with a mean CoV over the 69,815 peptides for all four alleles of 0.135 and a correlation coefficient over 0.988 for the single microarrays (Supplementary Figure 2).

For further validation of the microarray readout, the amino acid composition of the top 2% peptides with highest signal was compared to the amino acid composition of peptide binders as obtained from the IEDB for the HLA molecules where available. The results of this analysis are shown in Supplementary Figure 3 and confirmed an overall high consistency between the two with correlation coefficients for HLA-DRB1^*^03:01 and HLA-DRB1^*^15:01 above 0.910.

For further analysis, the median of the triplicate was used.

### Prediction of Microarray Data

**Figure 1:**
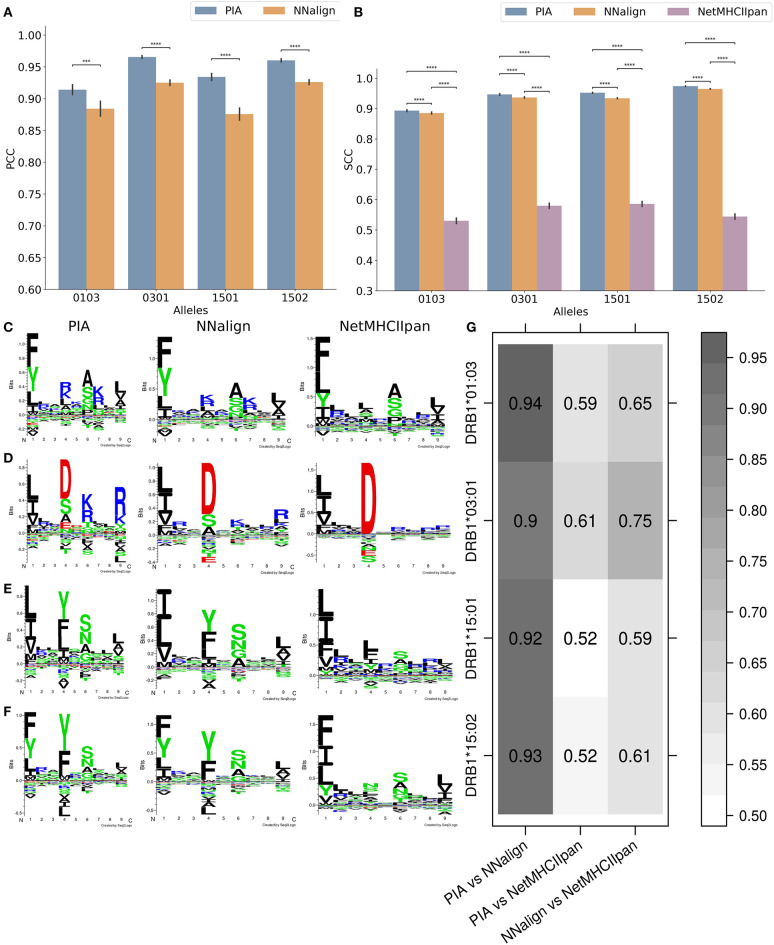
Performance of the models on peptide microarray data and resulting motifs. **(A)** The Pearson correlation coefficient (PCC) and **(B)** the Spearman correlation coefficient (SCC) on the independent test dataset of the peptide microarray are shown. The pairwise *p*-values were calculated using a non-parametric bootstrap hypothesis test with 1,000,000 bootstrap iterations. *0.01 < *p* ≤ 0.05, **0.001 < *p* ≤ 0.01, ***0.0001 < *p* ≤ 0.001, and ****p ≤ 0.0001. Motif plots of **(C)** DRB1*01:03, **(D)** DRB1*03:01, **(E)** DRB1*15:01, and **(F)** DRB1*15:02 based on the top 1% (from a pool of 100,000 random natural peptide) binding peptides generated with the deep learning model (PIA), NNAlign model and NetMHCIIpan-3.2 (8). **(G)** Pearson correlation coefficient (PCC) of the position specific scoring matrices (PSSM) between the different models.

For building the prediction models, the microarray data was log-transformed to reduce the skew and to optimize the range of the data. We trained the NNAlign and the GRU based PIA models using 10-fold CV for each allele. In all cases, PIA outperformed NNAlign. Figures 1A,B show the PCC and the Spearman correlation coefficient (SCC) performance values of the two models on the test dataset. Here, PIA outperforms NNAlign in all cases. Figure 1B also includes the SCC performance of NetMHCIIpan-3.2, which is trained on *in vitro* IC50 binding values demonstrating at least a SCC of above 0.53 for the different alleles for predicting the chip test data.

To quantify the consistencies between different data types and prediction models, binding motifs were estimated for each HLA molecule and prediction model (Figures 1C–F). The binding motifs identified by the peptide microarray based models are close to identical and in most cases similar to those generated with NetMHCIIpan-3.2 using Seq2Logo (8, 33). To compare the motifs obtained by the two microarray-based models, we performed a correlation analysis of the 9 × 20 position specific scoring matrix produced by Seq2Logo defining the predicted binding motif. In all four cases, we obtained PCC values above 0.90 (Figure 1G). When comparing the NNAlign and NetMHCIIpan motifs, the correlation values were still very high with 0.59–0.75.

### Predict Ligands Measured by Mass Spectrometry

**Figure 2:**
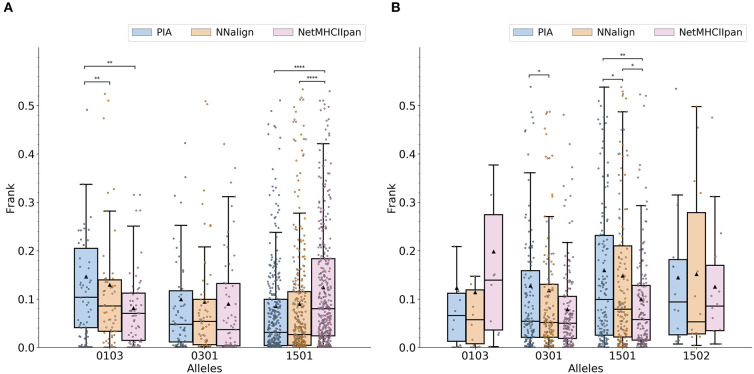
Comparisons of prediction quality on **(A)** MS ligand and **(B)** epitope data. The center line inside the box indicates the median Frank and the triangle shows the mean Frank. The data points available in IEDB are represented using a jitter plot. The colored box covers the interquartile range. The whiskers represent 1.5-fold of the interquartile range. Pairwise *p*-values were calculated using a Wilcoxon signed-rank test (applying Pratt's zero method). *0.01 < *p* ≤ 0.05, **0.001 < *p* ≤ 0.01, ***0.0001 < *p* ≤ 0.001, and ****p ≤ 0.0001.

To further assess the predictive power of the developed methods, we performed a benchmark on a set of HLA eluted ligands as obtained from the IEDB (7). Here, the Frank value was used as performance measure (8). In short, Frank is the proportion of peptides within a source protein with a prediction value greater than the given ligand. The Frank is 0 if the ligand is the peptide with the highest binding score and 0.5 for random predictions. To limit the effect of noise and falsely positive assigned data points, only ligands that obtained a Frank value of 0.15 or less for at least one of the included prediction models were included in the benchmark. As the IEDB currently does not contain any ligands for DRB1^*^15:02 the molecule was excluded from our analysis (Supplementary Table 1). The results (Figure 2A) demonstrate an overall comparable performance of the three methods. The microarray-based methods and NetMHCIIpan-3.2 each outperform the other for one dataset (NetMHCIIpan-3.2 performs better for DRB1^*^01:03, and PIA and NNAlign for DRB1^*^15:01). No consistent performance difference was observed between the NNAlign and PIA models.

### Predict CD4+ Epitopes

The same analysis performed on the HLA eluted ligand data was done on a set of CD4+ T cell epitopes available from the IEDB (Supplementary Table 1). The results (Figure 2B) show that the microarray-data based models in most cases performed on par with NetMHCIIpan-3.2. For DRB1^*^15:01, NetMHCIIpan-3.2 significantly outperformed both peptide microarray-based methods. For DRB1^*^01:03, the microarray-based models showed an increased performance compared to NetMHCIIpan-3.2. This latter difference was, however, not statistically significant due to the limited number of epitopes available in the benchmark. Moreover, the results indicate a slightly improved performance of NNAlign over PIA.

## Discussion

Genetic variants in the HLA gene region have been associated with a multitude of diseases, not only autoimmune conditions. Earlier work suggests this to be caused by an intrinsic property of particular HLA variants [for instance different HLA-DQ alleles influencing IL-17 production in T-cells irrespective of the peptide ligand (35)]. However, beyond this and for most HLA's and diseases, the detailed underlying mechanisms and candidate antigens remain unknown. Experimentally testing all possible peptide-HLA combinations to identify the relevant antigens for a given disease is a major undertaking, and with current technologies in most cases not feasible.

To deal with this limitation, we here present a new type of HLA-II antigen interaction assay based on high-density peptide microarrays. This technique allows the assessment of more than 200,000 independent peptide-HLA interaction tests within one single experiment.

We demonstrate how this high-density peptide array serves as a novel, valuable source for high-throughput and high-volume data to accurately characterize the peptidome of HLA-II molecules and its binding specificity. We demonstrated this by quantifying the consistency between internal replica (peptides analyzed multiple times on a given microarray), and by comparing the amino acid composition of the peptidomes as obtained from the peptide microarray to that obtained using conventional *in-vitro* binding assays with solid phase synthesized peptides. We furthered the validation by applying machine learning methods to mine and extract the HLA binding signal from the microarray data and compared the derived binding motif and power of the associated prediction model to state-of-the-art methods trained on conventional *in-vitro* binding data. All comparisons confirmed a high consistency of the microarray data with conventional methods.

In our study, two different machine learning algorithms were applied to mine the large-scale microarray datasets. The first is NNAlign, which is the basis for NetMHCIIpan-3.2 and NetMHCII 2.3 (8, 22) accepted to be among the best available for prediction of peptide binding to HLA-II (30). The second, PIA is based on GRU, a deep learning architecture developed for sequence learning. Both algorithms are able to capture the signal within the peptide microarray data and predict the microarray test dataset with very high performance.

Moreover, the two prediction models trained on the microarray data were benchmarked against NetMHCIIpan-3.2 on independent data of HLA eluted ligand and CD4+ epitope data obtained from the IEDB. Here, all models were found to perform at par, suggesting that the measurements obtained from the microarray are accurately capturing signals of peptide-HLA binding.

The microarray experiments performed here were conducted in the absence of HLA II peptide- loading chaperones such as HLA-DM and HLA-DO earlier demonstrated to play a role in editing the repertoire of HLA class II binding peptides (36, 37). Future work will tell if similar results are obtained in the context of the peptide-microarray technology.

Overall, our results suggest that the described microarray technology for large-scale evaluations of peptide-HLA-II interaction is accurate, precise and highly scalable. We believe this result opens a venue of novel applications addressing challenges and biological problems that can only to a limited extent be addressed using conventional immunoassays. Such applications include mapping the impact of peptide-specific post-translational modifications (such as phosphorylation, deamination, or citrullination, all of these modifications can be added in the peptide synthesis step, i.e., in the array design process) on HLA-II binding and unbiased large-scale screening for HLA-II binding of pathogen proteomes. The herein presented *in-silico* technology data is in our opinion a good addition to immunopeptidome data for the next generation of prediction tools.

## Data Availability Statement

The high density peptide raw datasets generated for this study can be downloaded from https://www.ikmb.uni-kiel.de/resources/download-tools/publicly-available-data. All other data are available from the corresponding authors upon request.

## Author Contributions

ER, TØ, SB, AF, and MW designed the wet lab experiments. TØ performed the wet lab experiments. MW prepared the data for training. HE implemented PIA. HG trained NNAlign and prepared the IEDB data, and plotted the final figures. MW, HG, FD, and MN performed statistical analysis. MW and MN wrote the paper with input from HG and HE. All authors commented on the final manuscript.

## Conflict of Interest

The authors declare that the research was conducted in the absence of any commercial or financial relationships that could be construed as a potential conflict of interest.
