# Supplementary material for: Unbiased Characterization of Peptide-HLA Class II Interactions Based on Large-Scale Peptide Microarrays; Assessment of the Impact on HLA Class II Ligand and Epitope Prediction
Source: Front Immunol. 2020 Aug 5;11:1705. doi: 10.3389/fimmu.2020.01705 (PMC7438773; doi:10.3389/fimmu.2020.01705)
Supplement: Supplementary file 1 [file Data_Sheet_1.docx]

Supplementary Material

# Supplementary Methods

## NNAlign

The final NNAlign model is an ensemble of 400 networks. The single networks are trained on one of the 10 cross-validation datasets with 10, 20, 40 and 60 hidden neurons and starting with 10 different initial configurations.

Each model was trained for 1200 cycles with a learning rate of 0.10 (eta), without early stopping. A burn-in period of 10 cycles was used where amino acid preference at position 1 is imposed (def: ILVMFYW). The peptide binding core length was set to 9 amino acids.

## Deep Learning Model PIA

PIA is a gated recurrent neural network (GRU) based model (1) that is composite of an embedding layer which projects each amino acid into a continuous representational space of eight dimensions, followed by a GRU layer with 100 units, followed by a batch normalization layer (2) and finally an output neuron giving the prediction score of each peptide. A sigmoid activation function was used for the final layer to restrict the output to be between zero and one.

The model parameters were optimized using Adam (3) to minimize mean absolute error between the predictions and the true labels. Training was carried out for 500 epochs with a batch size of 1024 using an Nvidia Tesla P 100 GPU.

As the models were trained using a 10-fold cross-validation (CV) dataset scheme, a final ensemble of the 10 models, one per CV dataset, with the highest SCC on the validation dataset was constructed. The output of the ensemble was computed as the mean.

# References

1. Cho K, Van Merriënboer B, Gulcehre C, Bahdanau D, Bougares F, Schwenk H, Bengio Y. Learning phrase representations using RNN encoder-decoder for statistical machine translation. *EMNLP 2014 - 2014 Conf Empir Methods Nat Lang Process Proc Conf* (2014) Available online at: https://arxiv.org/abs/1406.1078v3

2. Ioffe S, Szegedy C. Batch normalization: Accelerating deep network training by reducing internal covariate shift. *32nd Int Conf Mach Learn ICML 2015* (2015) **1**:448–456.

3. Kingma DP, Ba JL. Adam: A method for stochastic optimization. *3rd Int Conf Learn Represent ICLR 2015 - Conf Track Proc* (2015)1–15.

# Supplementary Figures and Table

## Supplementary Figures

**
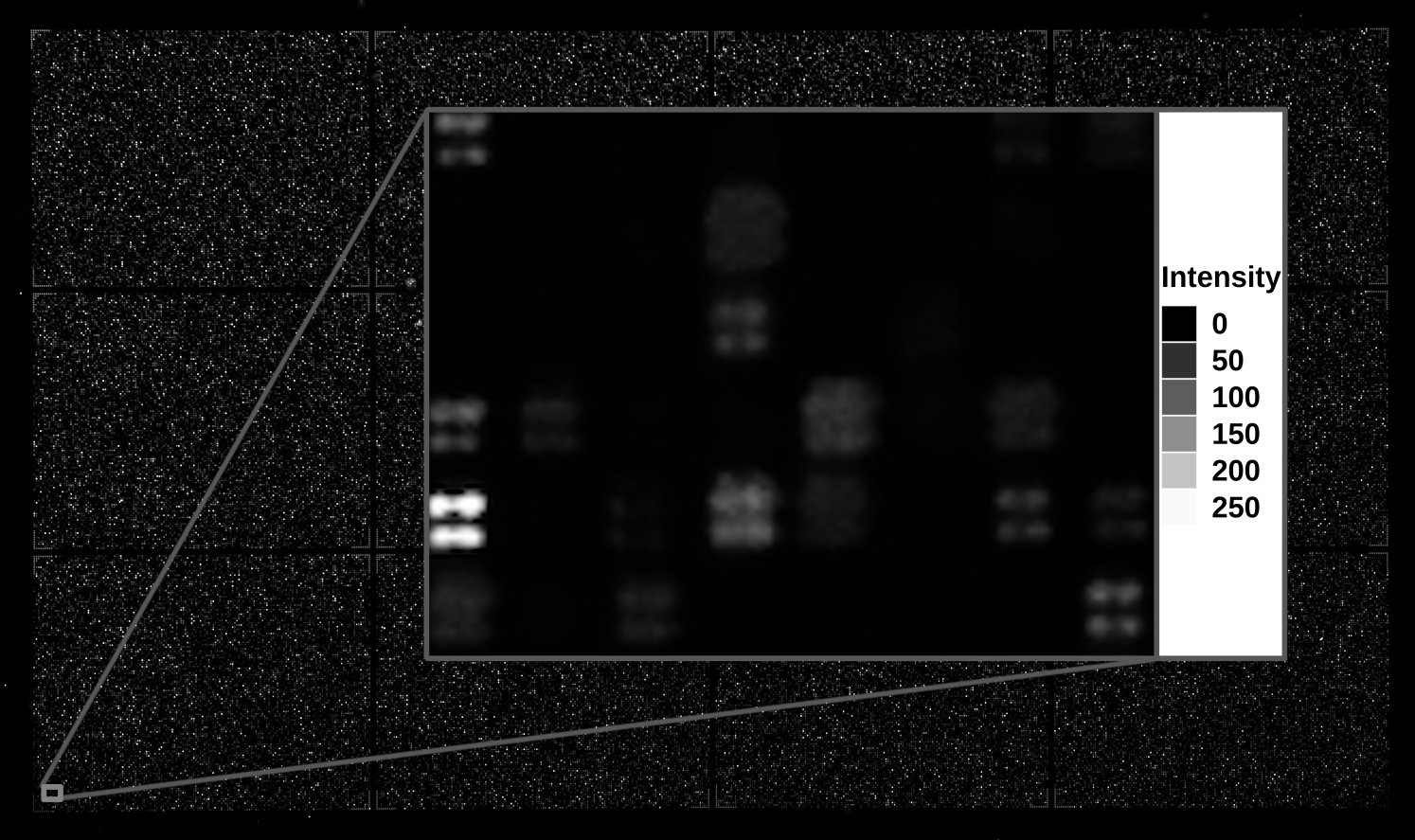
Supplementary Figure 1.** Exemplary peptide microarray raw readout. The background figure shows the complete readout with the twelve subfields separated through an empty region (black). The corners of the fields were marked with the CLIP marker peptide. As CLIP is a good binding peptide, the fields are visible as bright angles. Additionally, a zoom-in of a small area is shown. The peptides were synthesized in fields defined by 2 x 2 mirrors and a one-mirror-wide empty region separated the individual peptide fields.

###
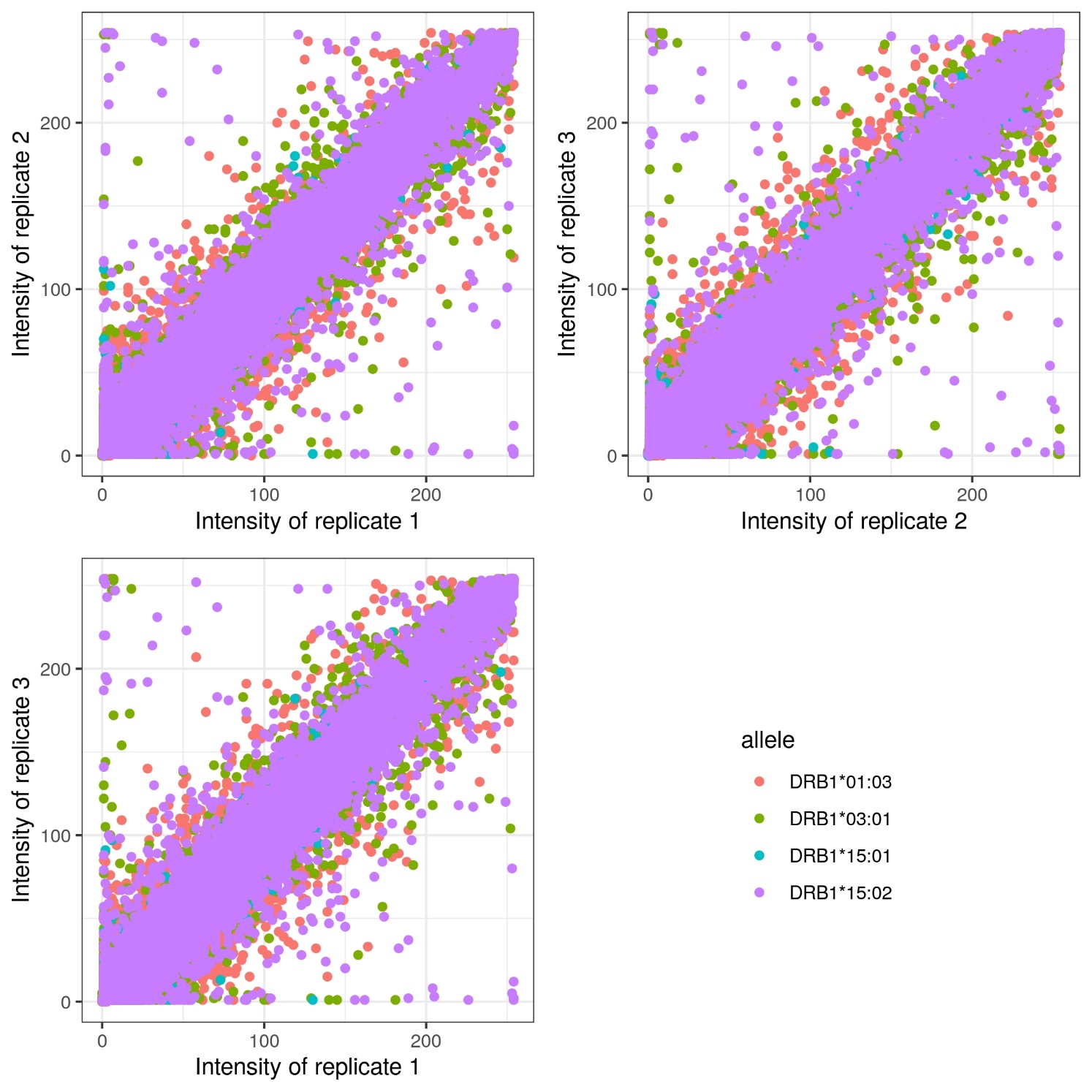


**Supplementary Figure 2.** Correlation of the three replicates. The measurements get their number of replicate randomly.

**
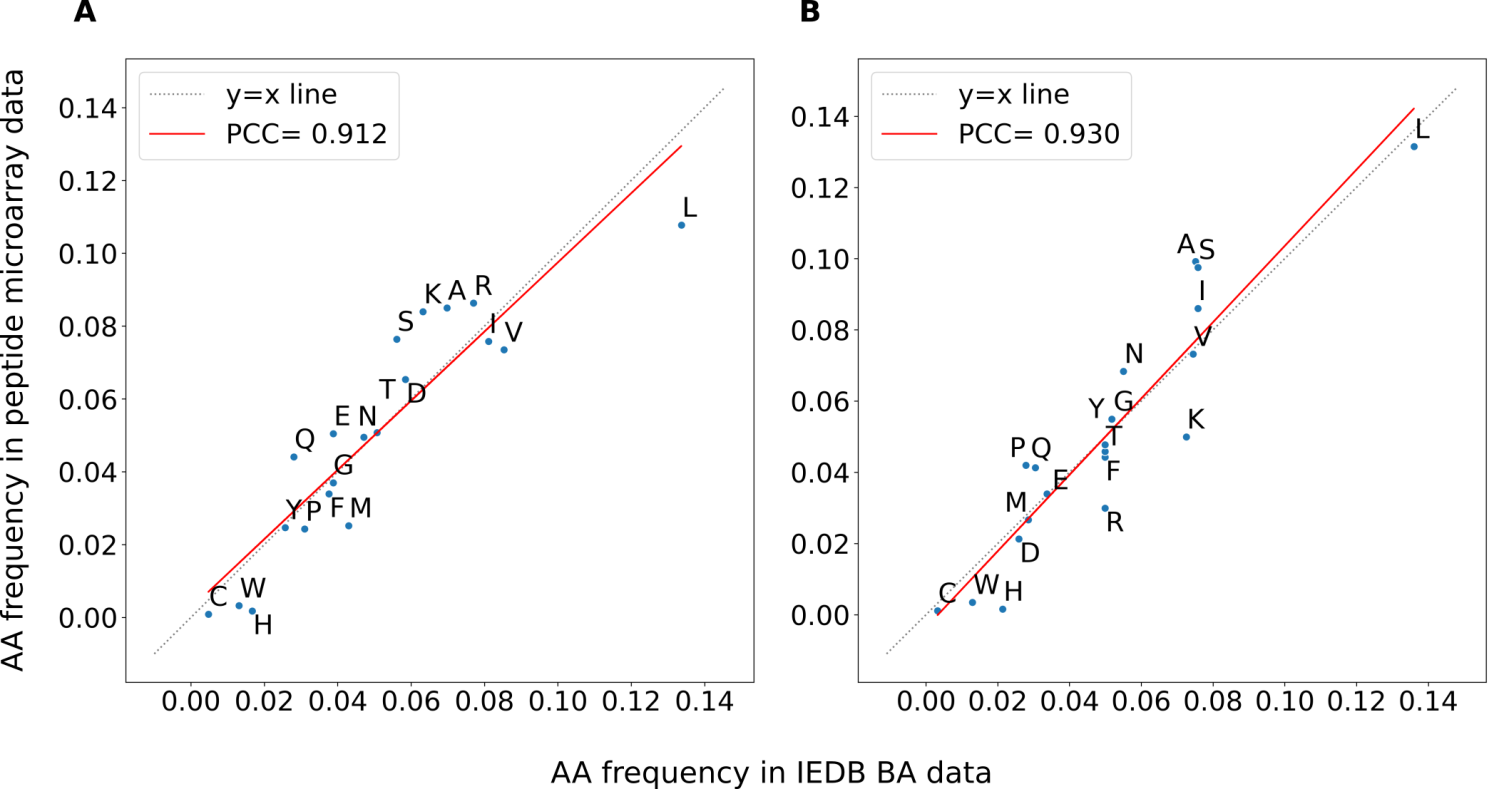
**

**Supplementary Figure 3.** Amino acid frequencies in binding peptides. Comparison of the amino acid frequency of the peptide microarray data against the IEDB binding affinity (BA) data for alleles DRB1*03:01 **(A)** and DRB1*15:01 **(B)**. The grey dotted line represents the identity line (y=x) and the red line is the fitted linear regression line (Pearson correlation coefficient shown in legend). Only the top 2% binding peptides from both data sources are included in the analysis.

## Supplementary Table

**Supplementary Table 1.** Summary of positive test data downloaded from IEDB. The numbers in brackets are those remaining after applying the Frank filter.

| DRB1 | *01:03 | *03:01 | *15:01 | *15:02 | Σ |
| --- | --- | --- | --- | --- | --- |
| Epitopes | 11 (10) | 224 (145) | 242 (152) | 25 (14) | 502 (321) |
| MS ligands | 126 (62) | 114 (65) | 479 (373) | 0 | 719 (500) |
